# “Your sexuality is yours and yours alone”: a reflexive thematic analysis of sexual orientation microaggressions and their impact on LGB emerging adults’ sexual health knowledge and attitudes

**DOI:** 10.3389/fpubh.2025.1522751

**Published:** 2025-03-03

**Authors:** Ty A. Robinson, Taylor M. Coleman, Chakema Carmack

**Affiliations:** ^1^Department of Psychology, University of Maryland College Park, College Park, MD, United States; ^2^Psychological Health & Learning Sciences Department, University of Houston, Houston, TX, United States; ^3^HEALTH Center for Addictions Research and Cancer Prevention - RCMI, University of Houston, Houston, TX, United States

**Keywords:** sexual and gender minorities, microaggression, sexual behavior, sexual health knowledge, reflexive thematic analysis

## Abstract

**Introduction:**

Lesbian, gay, and bisexual (LGB) adults often experience cissexism, heterosexism, and other forms of discrimination, which, as a result, leaves LGB adults vulnerable to identity-related victimization such as sexual orientation microaggressions (SOMs). These derogatory, hostile, and homophobic insults can lead to adverse mental and physical health outcomes for this minoritized group. While research has established taxonomies related to SOMs and their impact on the mental health and identity development of LGB people, little research has addressed the systemic influence SOMs have on sexual health knowledge and risk-related sexual behavior.

**Methods:**

The present study conducted four focus groups with LGB emerging adults (*N* = 17; *M* = 20.4 ± 2.4) to understand how their experiences with microaggressions might affect their knowledge and attitudes about sex and sexual risk behavior.

**Results:**

Reflexive thematic analysis resulted in four themes as critical aspects of (a) early familial moments, (b) religious persecution about their sexuality, (c) coping and resilience strategies and sexual health, and (d) sexual education and miseducation.

**Discussion:**

Results suggest that SOMs greatly influence how LGB individuals view their identity, sexual health, and attitudes toward sexual behavior. Additionally, addressing these microaggressions in the education, family, and healthcare systems may enhance healthcare access and quality and create inclusive environments that encourage equitable experiences early on in one’s identity development. Given that sexual health education begins during the stage of adolescence, future research can utilize these findings to design a study that understands LGB youth experiences of SOMs and how it impacts their identity development, well-being, and sexual health behaviors and attitudes.

## Introduction

Research has consistently shown that lesbian, gay, and bisexual (LGB) people experience disproportionate HIV and sexually transmitted infections (STIs) disease burdens due to the lack of affirming healthcare, sexual health education, and resources (1, 2, 59). Additionally, LGB people are vulnerable to experiencing multiple forms of microaggressions, violence, sexual assault, and harassment related to their sexual orientation and gender identity (3). Previous research by the U.S. Department of Justice National Crime Victimization Survey indicated that sexual minorities experience victimization at an increasing rate (4). From 2017 to 2020, LGB people were 2–7 times more likely to experience victimization (e.g., bullying, physical assaults) compared to cisgender heterosexual individuals.

A particular type of microaggression that has received increased scholarly attention is sexual orientation microaggressions [SOMs; Nadal (5)]. These intentional or unintentional derogatory, hostile, and homophobic insults have been linked to victimization (e.g., bullying and physical assaults) and adverse mental health outcomes among LGB people (6). Nadal et al. (7, 8) theoretically proposed a taxonomy of SOMs through focus group studies with LGB adults to understand their experiences. The taxonomy that was established included qualitative themes such as: (1) the use of heterosexist language, (2) endorsement of heteronormative culture and behaviors, (3) assumption of abnormality, (4) denial of the reality of heterosexism, (5) assumptions of uniformity among all LGB people, (6) the dehumanization of LGB people, and (7) disapproving LGB experiences, and (8) violent assaults against LGB people.

Subsequent research has sought to confirm or expand upon Nadal et al. (7) taxonomy by examining SOMs in varied environmental contexts such as primary school, higher education, and psychotherapy providers (9–14). From the psychotherapy perspective, where LGB people are the clients, Shelton and Delgado-Romero (14) sought to understand how LGB and queer (LGBQ) adults experience SOMs when engaging in psychotherapy, where the therapist (or medical provider) is the perpetrator. LGBQ clients shared their experiences of SOMs in therapy, where the psychotherapist pathologized their LGBQ identity as a source of their presenting problem, made stereotypical assumptions, attempted to overidentify with uncomfortable messaging, and expressions of heteronormative actions and bias. The authors were able to show how SOMs can occur at an interpersonal level with a medical provider and made additions to Nadal (7) taxonomy for this context, which informed psychotherapy education and the treatment of LGB clients.

Taking a systemic perspective, another example of a taxonomy of SOMs delved into understanding how the SOM experience of sexual minority Chinese youth in Hong Kong can inform an equitable sexual education curriculum and training for educators in their community (11). Furthermore, the participants who discussed their experience in current sexual education workshops stated that the curriculum focused on religious abstinence and preparation for “being a good wife.” Kowk and Kwok (11) understanding of how SOMs are present in institutional and primary school sexual education spaces can inform future curriculum development and trainings. However, there remains a gap in understanding how SOMs influence sexual health knowledge and sexual behaviors among racial/ethnic LGB people. In Table 1, we provide information on selected taxonomies from previous research.

**Table 1:** Key sexual orientation microaggressions (SOMs) themes in previous literature.

Author(s)	SOMs themes	Definition
Nadal et al. (7)	Theme 1: Use of heterosexist terminology	“Heterosexist language is used to degrade LGB persons.”
	Theme 2: Endorsement of heteronormative culture/behaviors	“LGB persons are expected to be or act like heterosexuals.”
	Theme 3: Assumptions of universal LGB experience	“Heterosexual individuals assume that all LGB persons and their experiences are the same.”
	Theme 4: Exoticization	“When LGB persons feel they are dehumanized or treated like an object.”
	Theme 5: Discomfort/disapproval of the LGB experience	“Overt discomfort from heterosexual people.”
	Theme 6: Denial of reality of heterosexism	“When a heterosexual individual denies that heterosexist or homophobic experiences exist.”
	Theme 7: Assumption of sexual pathology/abnormality	“LGB individuals have been thought to be oversexualized, sexual deviants, or both.”
	Theme 8: Threatening behavior	“Victims of assaults, threatening behavior, or both.”
Platt and Lenzen (13)	Theme 1: Endorsement of heteronormative culture	“Microaggressive statements that reflect endorsement of heterosexual behavior as normal and expected.”
	Theme 2: Sinfulness	“An underlying assumption that having a non-heterosexual orientation is sinful and morally deviant.”
	Theme 3: Homophobia	“Communicates irrational anxieties toward non-heterosexual individuals.”
	Theme 4: Heterosexist language/terminology	“These language microaggressions reflect the underlying negative assumption that being non-heterosexual is deviant and outside of the norm.”
	Theme 5: Oversexualization	“Microaggressive statements that reflect the negative stereotype that all non-heterosexual individuals are primarily interested in sex and physical gratification only.”
	Theme 6: Undersexualization	“Microaggression experiences that reflected a surface level acceptance of the individual being a sexual minority, but only when the person was not actively in a relationship:”
	Theme 7: Microaggressions as humor	“Microaggressive statements were delivered in a joking or humorous manner.”
Kwok and Kwok (11)	Theme 1: Approving heteronormative culture and invalidating non-heterosexuality	“Sexual minorities are expected to be heterosexual or to act as heterosexuals.”
	Theme 2: The use of heterosexist and abusive language	“Students described having overheard or personally received comments that were obviously offensive to LGBQ individuals.”
	Theme 3: The assumption of sexual abnormality: endorsing stereotypes	“The endorsement of stereotypes and sexual prejudice wherein some educators and students portrayed sexual minorities as morally, and/or emotionally, unnatural.”
	Theme 4: Allowing institutionally endorsed microaggressions	“Microaggressions came not only from individual students, teachers, school social workers, or administrators but were also expressed through school surveys, leadership, or teaching curricula.”

Studies have also demonstrated that SOMs are linked to negative mental health outcomes, including heightened anxiety (15, 16), depression (17, 18), suicidal ideation (19, 20), post-traumatic stress disorder symptoms (21, 22), and alcohol misuse (23–25). Responses to SOMs can manifest behaviorally (e.g., passivity, confrontation), cognitively (e.g., resilience, conformity), emotionally (e.g., anger, sadness, embarrassment), or through distancing from support systems (25, 26).

In terms of minority stressors, the literature suggests that experiencing SOMs can lead to internalized homophobia (27, 28), potentially increasing risky behaviors, including condomless sex, thereby heightening HIV risk (23, 29). As sexual health encompasses physical, emotional, mental, and social well-being (30), recognizing the impact of SOMs on LGB sexual health is critical, especially given that current social discourse on sexual health often minoritizes LGB experiences while centering on heterosexual norms (31, 32).

## Critical constructivism

Taken together, previous studies highlight the complex nature of sexual health (e.g., sexual health knowledge), sexual health outcomes (e.g., disproportionate STI rates), and mental health as they relate to SOM experiences for LGB persons. However, it is currently unclear as to how SOM experiences within LGB populations impact their sexual health knowledge and how they make sense of experiences surrounding this while also considering intersectional identities. Given that the taxonomy of SOMs represents an emerging area of research, there is a need for deeper insights to identify and explore thematic narratives through the experiences of LGB adults.

Given the ways in which critical constructivism emphasizes how knowledge and perspectives are shaped by social pressures (33), it served as the theoretical foundation for this present study. As a philosophical paradigm, critical constructivism provides a foundation for meaningfully understanding the knowledge and experiences of others. This may be done through the interactions of culture, institutions, and history. Knowledge is shared and shaped through contextual experiences and interpreted through meaningful dialog. Within a critical constructivism theory, reflection and reflexivity are emphasized. Leaning heavily on Kincheloe (34) epistemology of critical constructivism, the present study (and its authors through the use of reflexive thematic analysis), recognized that (a) knowledge is socially constructed; (b) the importance of emotion, in addition to logic, in the production of knowledge, (c) the “knower” (i.e., the researchers and their perspectives) and the “known” (i.e., truth in evidence) are invariably interconnected, (d) understanding the existence of multiple realities and the humility needed on the researchers’ part in understanding the perspectives of oppressed people, (e) the intersection of personal experiences and various existing worldviews, and (f) the respectable criticism of traditional positivism and reductionistic methods of reliability.

### Present study

The present study focused specifically on the sexual orientation of persons who have same-sex attractions and practiced/intend to practice same-sex coitus: LGB young adults. Transgender and queer individuals represent gender minority identities (−although “queer” is often colloquially used to indicate gender identity or sexual orientation). Although transgender and queer individuals may have same-sex sexual attractions, gender identity was not central to the present study. This study was developed and carried out as a reflexive thematic analysis, whereby the researchers generated themes (from qualitative participant data) as interpretive stories with a uniting meaning (35). Here, we utilized reflexive thematic analysis using phenomenological framing. Notably, reflexive thematic analysis is concerned with the study of lived experiences and subjective sense-making; coupled with phenomenological framing which seeks to explain the nature of things through the people who experience them. In utilizing a reflexive thematic analysis, the framework provided flexibility in connecting data of seemingly unrelated dialogs to an evident central meaning (35). Thus, the researchers of the current study explored the nature of SOMs experienced in a diverse sample of LGB young adults as they related to their sexual health behavior and knowledge. The researchers also recognized that participants may hold other marginalized facets of identity in addition to their sexual orientation (e.g., race/ethnicity and gender) that may intersect with their experience of SOMs.

Thus, the overarching research question that guided the study was, “How have your experiences of sexual orientation-related microaggressions influenced your knowledge and beliefs about sex and sexual health?” To address this, the purpose of this study was to understand the lived experiences of young adult LGB individuals going beyond previously established taxonomies of SOMs. The taxonomies described above, in effect, are categorizations of the types of SOMs commonly experienced by LGB individuals. We did not intend to validate these previously established taxonomies, but to add an additional understanding of how SOMs shape the sexual health knowledge and/or behaviors of LGB people. Additionally, we hoped to establish a foundational taxonomy of SOMs related to sexual health and behaviors that may differ and/or be similar to previously established taxonomies.

## Method

### Recruitment, participants, and procedures

Recruitment for the current study occurred from August 2021 to December 2022 on a university campus through flyer postings, the LGBTQ Resource Center at the university, community partner events, and an online research participation platform. Potential subjects were screened prior to the start of the study. Eligibility criteria included: (1) self-identified as lesbian, gay, or bisexual; (2) adult aged 18 years or older; (3) English speaking; and (4) not currently pregnant or lactating. The university’s Institutional Review Board (#00001565) approved the study’s procedures.

Participants were given a brief demographic questionnaire. The study comprised *N* = 17 participants ages 19–26 (*M* = 20.8; *SD* = 1.7). Participants self-identified as cisgender female (*n* = 10, 59%), while the remaining 41% identified as cisgender male (*n* = 7). Of the participants, 41% (*n* = 7) identified as Black/African American, 35% (*n* = 6) as Hispanic, and 24% (*n* = 4) as white. Regarding sexual orientation, 41% (*n* = 7) of the participants identified as lesbian, 29.5% (*n* = 5) as gay, and 29.5% (*n* = 5) as bisexual (see Table 2).

**Table 2:** Sociodemographic characteristics.

Participant (Pseudonym)	Age	Gender identity	Race-ethnicity	Sexual orientation
Suri	19	Cisgender woman	African American	Lesbian
Liza	20	Cisgender woman	African American	Bi-sexual
Imani	20	Cisgender woman	African American	Bi-sexual
Bellamy	25	Cisgender woman	African American	Lesbian
Christian	22	Cisgender woman	Hispanic	Lesbian
Jennifer	19	Cisgender woman	Hispanic	Lesbian
Amelia	21	Cisgender woman	Hispanic	Bi-sexual
Sarah	21	Cisgender woman	White	Lesbian
Korin	20	Cisgender woman	White	Lesbian
Shawn	20	Cisgender woman	White	Lesbian
Jason	24	Cisgender man	African American	Bi-sexual
Corbin	23	Cisgender man	African American	Gay
Kelvin	19	Cisgender man	African American	Gay
Chris	20	Cisgender man	Hispanic	Gay
Ricky	21	Cisgender man	Hispanic	Gay
Donaldo	20	Cisgender man	Hispanic	Gay
Rod	20	Cisgender man	White	Bi-sexual

Four focus groups were conducted in person in a closed-door classroom at the university (three focus groups consisted of *n* = 4 participants, and one consisted of *n* = 5 participants). Upon consenting, participants were notified that they would be digitally audio-recorded throughout the focus group session. Each focus group contained two trained moderators who underwent facilitation training with the senior author and were all experienced in following Krueger (36) best practices for focus group facilitation. The facilitators and participants introduced themselves and were told they could use an alias name for anonymity. To facilitate discourse surrounding SOMs and issues of sexual health, the participants were guided throughout the discussion using a focus group script developed to address various aspects of the overarching research question.

Due to the reflexive nature of the study, conversation flowed freely and openly, and the trained moderator was skilled in keeping a balance that explored various aspects of SOM experiences (guided by the script) while allowing participants to collectively explore any of the aspects in depth, as the conversations dictated. An assistant moderator took live notes of the sessions, denoting emphasized parts of the conversation (e.g., mutual agreement among the participants of a particular point of view) and non-verbal cues (e.g., head nods) (36). Upon the conclusion of each session, the participants were asked if they had anything else to share and were thanked for their participation. The sessions lasted for approximately 1.5 h, and both the lead and assistant moderator believed that participants could freely express all the points validly to the experiences they wanted to share.

### Researchers’ positionality and social context

Reflexive thematic analysis embraces the researchers’ subjectivity as a resource for research (35). Therefore, it is important to note how our identities, privileges, and oppression influence our study design and methodology (37). This paper’s authors represent various identities related to race, ethnicity, gender, sexual orientation, and individual experiences of oppression. As we interpret the data from a phenomenological perspective, it is crucial to pontificate how our history and experience contribute to the results and interpretations of this study. Each author provided their perspectives throughout the research design and analysis. During critical parts of the process, particularly the data interpretation and theme generation, the authors met to discuss how these “stories” may relate to *their* lived experiences and how that may influence the reflexive thematic analysis presented herein. Thus, the authors would like to acknowledge aspects of their identity and social background.

#### Ty A. Robinson, Ed.M.

The first author identifies as a Black, gay, cisgender man and is a counseling psychology Ph.D. student: As a contributor to the writing of this manuscript, it is important to state that I am a Black gay cisgender man who was born and raised in the southern region of the United States (U.S.). Much of my professional experience has focused on providing health, wellness, and educational services to communities of color and engaging in service within the field of psychology. My educational and personal journey has been faced with obstacles related to the discriminatory, racist, and Eurocentric practices in the academic system. With this, I drew upon my past and present experiences to push the needle forward in addressing systemic racism, oppression, and discrimination to, in turn, create meaningful change that can result in the healing and liberation of communities of color and sexual minorities. Throughout this study, I reflected upon my experiences when conceptualizing how our participants were experiencing discrimination, coping, and reacting behaviors. I acknowledge that my position and experiences have contributed to the transcription, coding, and interpretation of results.

#### Taylor M. Coleman, M.A

The second author identifies as a Black, heterosexual cisgender woman, and is a counseling psychology Ph.D. student: As a contributor to the writing of this manuscript, it is important to state that I identify as a cisgender, heterosexual, African American woman. I was born and raised in the southern United States, where I faced numerous challenges navigating systemic racism and microaggressions in both educational and professional settings. My lived experiences, coupled with my academic training, have provided me with a unique perspective that allows me to connect deeply with the focus group participants in this study. As a counseling psychologist in training, I have dedicated my career to understanding and advocating for marginalized communities, and much of my research has centered on the psychosocial experiences of individuals of color. I drew upon my own history of encountering microaggressions and discrimination to empathize with our participants and to ensure their voices were heard and represented accurately. This positionality allowed me to remain attuned to the nuanced ways in which microaggressions impact individuals’ mental and emotional well-being. My commitment to fostering a supportive and understanding environment for our participants influenced both the facilitation of the focus groups and the interpretation of the findings. Ultimately, I acknowledge that my positionality shaped my role in the research process and influenced the ways in which I analyzed the coping strategies and reactions of our participants to their experiences of discrimination.

#### Chakema Carmack, Ph.D

The third author identifies as a Black, heterosexual cisgender woman, and is an associate professor in the counseling psychology Ph.D. program: As a contributor to the writing of this manuscript, it is important to state that I was born and raised in the southern region of the United States and studied as an adult scholar in various regions of the country. I am a trained community psychologist, and my early training focused on psychosocial theory validation for diverse populations. I have been involved in HIV prevention research and the psychosocial aspects of sexual behavior for over 15 years. I’ve facilitated focus groups on various aspects of sexual behavior and intention as they relate to condom use and STI prevention behaviors and beliefs. Regarding the present study, I drew upon my scholarly mission to create meaningful change for communities of color that fosters a culture of sexual health. Throughout this study, I reflected upon my knowledge and comprehension of previous research regarding LGB populations, as well as interactions with LGB young adults in social and research settings to conceptualize how our participants experienced discrimination, coping, and reactivity. I acknowledge that my positionality and experiences have contributed to the purpose, coding, writing, and interpretation of the present research.

### Data analysis

The researchers applied thematic analysis following Braun and Clark (35) framework for reflexive thematic analysis which is valuable for comprehending participants’ unique experiences and mitigating potential research bias (38, 61). Specifically, reflexive thematic analysis was preferred due to potentially sensitive nature of our topics of interest (sexual orientation microaggressions and sexual risk behaviors) because it offers greater accountability in the researchers’ interpretation of the participants’ lived experiences. Additionally, flexibility in the reflexive thematic coding process allows for codes to change and evolve as the researcher attempts to develop meaning among the interconnected data points (35). The reflexive thematic analysis data analysis process consisted of six phases: (1) familiarization of the data, (2) systematic data coding, (3) generating initial themes, (4) developing and reviewing themes, (5) refining/defining and naming themes, and (6) reporting.

Three members of the research team listened to each focus group carefully several times to grasp the content and flow of discussions. The lead researcher (third/senior author) transcribed each audio recording verbatim and then read each transcript along with the audio recording for verification. Transcription data was entered into Atlas.ti, a qualitative analysis software in preparation for the first phase of familiarizing the data. Familiarization entailed reading through each transcript and notation. During notation, the researcher took notes of pieces of dialog that were of potential interest and the researcher’s response to that piece of dialog. In examining each piece, the researcher noted any assumptions that may underline or influence the researcher’s response or implications (e.g., an underlying belief that attitudes influence behavior). The researcher noted what was familiar or unexpected and reflected on any insight into their initial reaction to it.

We used a reflexive approach based on ‘organic’ coding, which takes a non-positivist approach (35, 39). The coder (lead researcher) engaged in both semantic and latent coding (35). A list of 26 codes was identified along with their corresponding dialog. Codes were compiled in a single list. The coder examined this list alone to ensure that it sufficiently captured the essence of the data. The research team examined the codes and dialog, and each reflected upon their assumptions. As this is a reflexive approach, a few guiding questions helped shape our discussion in preparation to create themes (40): What worldview did the code/dialog reflect; Were the meaning of codes/dialog located within the participant’s world (e.g., a direct reflection of what the participant thinks) or ‘out there’ in the social world; What did it make you think? Through robust notetaking and conversations among the research team that incorporated their perspectives and experiences, the research team clustered similar codes and examined the dialog to generate themes. Thus, within the coding process, meaning is generated as a co-constitutional interpretation at the intersection of the researcher, the research question under investigation, and the participant experience. Reflexive thematic analysis does not typically lend itself to a strict set of codes for the coding process; nor does it lend itself to positivist metrics of traditional thematic analysis such as quantifying codes or high inter-rater reliability. As inter-rater reliability is essentially a metric of the consistency and agreement of raters, it is not within the scope of *reflexive* thematic analysis to demonstrate this due to the inherently subjective and interpretive nature of the analysis on the part of the researchers (40, 41). In other words, as reflexive thematic analysis is concerned, this subjectivity is a resource for the research findings and not a hindrance. Although inter-rater reliability is not central to the reflective thematic analysis, this was mitigated by coding “checks” where the researchers discussed how different interpretations arose from our different perspectives when examining the created codes and dialog, and where applicable, combined perspectives for a richer, more nuanced understanding.

Five initial themes were created and were unanimously agreed upon by the research team. In generating these initial themes, the research team considered the theme’s quality in presenting novel information, its boundaries (e.g., too wide-ranging or not encompassing enough), and whether there was enough meaningful information to support the theme. A thematic visual was created to refine the themes. Taking a phenomenological approach, we also discussed how the dialog within each theme, or lived experience story that it represents, may be connected to another piece of dialog/participant’s story/theme. The research team reviewed the initial five themes by checking them against the coded data and revisited the transcribed data for congruency. As this fourth phase can be an iterative process, the research team believed two themes were best subsumed into one. Ensuring that they captured mutually agreed upon meaning, this resulted in four themes. Theme development concluded in refining, defining, and formally naming themes. The research team collaboratively wrote, edited, and re-wrote the commentary that will constitute the results below. We decided on the order of themes, selected the most demonstrative examples of the theme, and identified patterns across the themes and data extracts.

## Results

The themes generated illustrated the ways that the participants meaningfully reflected upon how their microaggression experiences contributed to their knowledge and beliefs about sex and sexual health. Through their lived experiences, these moments, or dialog pieces (data points), reflected the interwoven aspects of (a) early familial moments and (b) religious persecution about their sexuality, (c) coping and resilience strategies to address their emotional reactions to SOMs, and (d) attitudes/beliefs about risky behavior that shaped their education, or in some instances miseducation, about sexual health. The bracketed letters following participant pseudonyms indicate their self-identified sexual orientation: [L] lesbian female, [G] gay male, [Bm] bisexual male, or [Bf] bisexual female.

### Early familial SOM messaging

Participants discussed specific microaggressions targeted toward their sexual orientation as children even before they, themselves, could fully understand their sexual orientation. They explained both blatant slurs and subtle disrespect by parents, extended family, community figures, and school authorities. Family members often dismissed “signs” of their gender fluidity and developing sexual orientation as growing pains or an aspect of their behavior that they will one day “outgrow.” Participants reflected on how their family and friends assumed that their sexual orientation was an adolescent “phase” that they would soon grow out of. As Rod explained,

“I heard my mom tell her friend that my blue nail polish and eyeliner was because I was at that rebellious age, so don’t pay it any attention.” – Rod [Bm]

Some participants recalled childhood events of understanding their non-heterosexuality and being shunned with homonegativity by family members and other influential adults (e.g., teachers). As Christian discussed:

“I told my mom I had a crush on this girl in middle school. Like I was so in love with her. Like more than just a friend. I can remember her laughing and saying, ‘oh, it’s just because she’s your best friend; you both’ll be crushing on boys next year.’ It kinda confused me, cause I knew I never liked boys ‘that’ way.” – Christian [L]

Suri discussed the impact that family members attempting to alter their sexual orientation had on them as well.

“My little cousin, he was turning around, and he was jumping saying, ‘I’m the princess, I'm gonna be the princess,’ because he wanted to be rescued by the prince. And my aunts were yelling at him like, ‘No you’re the prince! You’re the prince!’ But I was different in the same way, so I knew what he meant.” – Suri [L]

Moderator notes indicated the non-verbal head nods of affirmation and verbal agreeance from other members in the group. Many participants understood this sentiment. To their understanding, when they started having physical attractions to their same sex-gender counterparts, family members would discourage non-heteronormative behavior. This contributed to further feelings of confusion and sadness about their *difference* sexually, particularly at a developmental time when acceptance and belongingness are important to healthy mental health development.

### Religious persecution SOMs (sub-theme: intersection of race/ethnicity)

SOMs related to religious persecution were identified through specific codes, including, church experiences, immoral homosexuality, and religion. Participants shared their experiences of facing discrimination within their religious community due to their same-sex attractions at an early age and throughout their adolescence. Some highlighted instances where religious persecution or religious pleading attempted to change their sexuality as if it were modifiable. As Jennifer and Corbin recalled:

“It makes you wonder why God made you this way when you’re a kid. I wanted to be less attracted to guys and tried for a while.” – Corbin [G]

“I became less interested in the church stuff around the time I was a junior or senior [in high school], because I couldn’t get over the fact that God actually cares who I’m attracted to. The way it was presented to me, I was confused… they said, this is a lifestyle choice and is wrong to do.” – Jennifer [L]

Participants who participated in religious activities discussed how homosexuality is viewed as a “heavier” sin than other common sins (e.g., lying, stealing, etc.). Consequently, this early experience of religious persecution significantly discouraged their continued participation in religion. Perceiving a God that is believed to love and nurture unconditionally caused confusion when contemplating that God would condemn them solely because of their sexuality. Their church institutions shaped their perception of these early experiences and typically involved immediate and extended family, which may be interconnected with early familial SOM messaging. Additionally, SOMs may be present within institutional structures such as churches, strengthening their salience within families that engage in regular religious activity.

“I was always brought with: God created Adam and Eve, NOT Adam and Steve or Lisa and Eve. It was always like that.” Liza [Bf]

Participants also shared their experiences with religious persecution as it related to their racial identification. Some African American participants reflected on how they perceived the African American church specifically felt about sexual orientation identification. One participant recalled very different experiences attending his African American church compared to attending his friends’ predominantly White church.

“The Black churches are definitely harsher [about accepting gay and lesbian persons]. They didn’t like how I expressed myself. Even as a kid, I’d get told to calm down or tone it down. I remember going to my friend’s methodist youth church, and they were way more welcoming and let me be me. …It was mixed race [-meaning multiple races attending].” – Kelvin [G]

“…This is why I don’t participate in the church. Black churches seem to think you’re going to some special, extra hot hell just because you’re not hetero. They’re so behind.” – Jason [Bm]

Although Kelvin’s experience was anecdotal, for example, it points to an early experience of institutional microaggressions that shaped his views of his church, his religion, and spirituality within his cultural group. Most Western religions are heteronormative in many aspects, and there seemed to be an emphasis on the Black churches not being approachable or inclusive of sexual orientation minorities. Notedly, participants of other races and ethnicities did not mention this within the study sample.

### Reactions to SOMs (sub-themes: societal norms and coping/resilience)

Emotional reactions to SOMs included various negative psychological affects such as anger, apathy, confusion, and self-repression. Participants shared how they reacted to SOMs at different life stages, from formative childhood to adolescence and college. Confusion and apathy were present as an emotional reaction to their experiences of religious persecution SOMs. Other negative reactions can be gleaned from various SOM experiences. Participants discussed their reactions to offensive microaggressions in school, work, and social/collaborative environments.

“I recently got mad in [university] class, because someone in our group did not want to do our group project on LGBT populations because she said, ‘please, no gay stuff.’ Talking about she’s ‘tired of everyone getting on the gay bandwagon.’ Then she said, ‘sorry I don’t want to offend nobody,’ but I was already offended.” – Amelia [Bf]

“I still get mad when I hear someone say, ‘That’s gay’ or ‘No homo’… Like why is gay and homo a diss [disrespect]?! I don’t go around saying ‘That’s straight’ or ‘No hetero’ to diss something!” – Chris [G]

A particular SOM could produce different emotional reactions in two different people. Presumably, these individual differences may be influenced by individuals’ personal histories about their sexual orientation and their awareness of it. Nonetheless, early familial experiences may also provide a buffer to negative emotionality about the heteronormative society. As another participant said in response:

“My dad told us [self and siblings] he was gay when we were in high school, so we’ve always been open when it comes to stuff like that. So it doesn’t bother me.” – Suri [L]

“I’ve always had thick skin, but what hurt my feelings the most was probably, you know when the preacher clearly said during the sermon one Sunday, ‘you’re going to hell or getting HIV!’ or whatever. But my mom said it’s not true.” – Jennifer [L]

In tandem with their reactions to SOMs, the issues of coming out became apparent. Some participants reacted to SOMs by denying their sexual orientation and keeping their emotions secret, while others grappled with internal anger and avoided social group activities if everyone knew they were gay. Societal norms seemed to be a form of SOM for them as maintaining their heteronormative social expectations became a priority above being their authentic self.

“So, this lady told my mom that she saw a ‘homosexual spirit’ on me, and it freaked me out. Cause I wasn’t ready to tell my family. And when she said that, I just shut down; like I was in denial. I was like no, no I’m not.” – Ricky [G]

“I felt like I lived a double life. Everything was good on the outside, basketball, soccer; but I think I was depressed about it [in junior high school] at the time and didn’t know it” – Jason [G]

Despite the negative emotionality surrounding disrespectful microaggressions about their self-identified group and the isolating feelings about being outed, participants discussed how they tended to cope with the negative emotionality, either mentally or behaviorally. Coping strategies and resilience in response to SOMs were largely positive. Participants spoke about the benefits of friendships, LGB issues – ‘then versus now,’ and hopefulness for the next generation. While some participants mentioned the supportive role of their parents or other family members, most emphasized the significance of relying on relationships with friends for support. They shared how friendships, particularly in high school and college, helped them cope with homophobia and gain acceptance of their sexuality. Being open to sharing was a sentiment shared by most of the LGB participants. As Imani advised:

“If you have a lot of trouble reconciling with your identity, it’s okay to ask other gay people about their experiences too, and that’s one thing I like to do is talk to other gay people about their experiences, don’t be afraid to reach out.” – Imani [Bf]

“Girls would make fun of me because I was always boyish, in how I dressed and carried myself. But I did have good friends. They would say, ‘forget them,’ or ‘who cares.’ It wasn’t in a dismissive way, though. I started to think, well if my straight best friends don’t care, I’m not going to care what haters think about me either. It was a good thing.” – Sarah [L]

Having a healthy, accepting friend group was a vital part of coping. For example, bullying situations in grade school and high school were buffered with both self-identified heterosexual and homosexual friends. In other words, regardless of their friends’ sexual identity, friends seemed to instill hope and support in their identity, which had positive impacts on their resilience to SOMs. Participants were asked about ideas about sexuality and sexual orientation that they would pass on. Responses centered around their resilience to embrace their sexual orientation in a positive way and the rejection of stereotypes present in SOMs.

“I know whether I have children, adopt or foster or whatever, they’ll certainly know that your sexuality is yours and yours alone.” – Donaldo [G]

“My mom put me in debutants. So they started these classes…All the information had to do with courting boys. My mom didn’t even ask me, she just put me in it. I mean, I want children. But I’m going to really find out who they are, so I can do things that fit *them*.” – Christina [L]

“They always just thought I was rough around the edges, you know, a tomboy or something. That’s why I find myself correcting my little cousins. Well, not correcting, but you know, opening their minds to different views about that.” – Sarah [L]

Debunking SOM and gender stereotypes for themselves in adulthood meant that these stereotypes would not be propagated within their social circles, nor would they be passed on to the next generation. LGB participants in this study conveyed a sense of hopefulness for the next generation regarding not propagating SOMs. Understanding the psychological trauma of their SOM experiences provided them with empathy and empowered them to take action to create a better, more positive social environment for others exposed to SOMs, regardless of their sexual orientation, particularly in younger generations. Many of the LGB participants spoke about wanting children, which is now acceptable in our society and seemed to be encouraged within their LGB community. Understanding that their sexual orientation was evident to them, even before they knew what it was, essentially debunks the erroneous lifestyle choice argument and made them malleable about sexual orientation labels and expectations they put on others. Being homosexual or bisexual in a heteronormative society allowed them to be more open and accepting of their future children’s sexuality, regardless of gender orientation and sexual orientation.

### Sexual health education and miseducation (subtheme: sociocultural influences on attitudes toward risky sexual behavior)

Participants were asked about how SOMs affected their knowledge of sexual health (e.g., STI knowledge, LGB STI health disparities, myths about sexuality in the LGB community) and decision-making about their sexual behavior. Conversations involved education about sexual health, HIV/AIDS, and disease and death, as well as miseducation about gay sex and promiscuity within the LGB community. When asked about how these experiences and SOMs shaped their knowledge of sexual health, participants spoke about HIV/AIDS as a “gay man’s disease” and how social media oversexualizes homosexual people and portrays them as excessively sexual.

“I was told that HIV originated from gay men in the 70’s. A teacher pulled me to the side one day and told me this. She said that gay people are dying because they are having sex. She knew I was gay -but actually, I’m bisexual- so she was basically telling me it’s ok to have feelings like that, but think twice before acting on it. Like, don’t ever have sex. She was being nice and didn’t know any better, but now that I’m grown, I’m like, ‘why can’t I enjoy same-sex sex just like everyone else enjoys sex?’” – Iman [Bf]

“In sex ed, we were taught that sex is for procreation only; man and woman; procreation only. But we all watch grown-up movies and we’re not stupid. Two girls [having sex] were not trying to procreate!” – Bellamy [L]

Participants discussed differences in gender norms related to the acceptance of homosexual cisgender males versus homosexual cisgender females. Some of the SOMs that contributed to their sexual education and miseducation assumed different “facts” dependent upon whether the “fact” (stereotype) was targeted toward gay men or lesbian women. The level of sexual education and the severity of miseducation about sexual health varied among the participants across the focus group sessions. Some participants understood sexual health and wellness and its relationship to their sexual orientation. However, there were also misconceptions about sexual disease acquisition.

“A lot of people are ignorant about what you could catch orally; you know, if you’re getting [oral sex] from someone and then hook up with someone else, you could pass something [STI] to somebody else.” – Rod [G]

“I hear people saying bisexual men are a lot more promiscuous than others which I have to disagree with.” – Jason [Bm]

“I was told gay men get more diseases because of, you know, anal sex. And lesbians have the least diseases because they don’t really do all that stuff.” – Jennifer [L]

“I don’t even think my doctor even knows I’m gay.” – Shawn [L]

In discussing sexual health education, the authors developed a sub-theme of SOMs and information related to sociocultural influences that interacted with how they processed sexual health as an LGB person. Attitudes toward risky sexual behavior such as condoms/prophylactics and drugs/alcohol were influenced by sociocultural influences. Rod recalled:

“As a teen, I gravitated to the rave culture, and it had me thinking that sex and drugs were ‘the life.’ But that’s only because there was nowhere else we were accepted. It’s like everyone on the fringe was all lumped together. Back then, gay was the ‘fringe’ culture.” – Rod [G]

Participants shared insights into various factors influencing their attitudes regarding risk-related and protective sexual behaviors. Contextual influences that shaped their attitudes about engaging in protected or non-protected sex were highlighted, such as rave cultures and the use of “hook-up” apps. The rave community in some regional areas served as a sub-community that influenced LGB attitudes about risky sexual behavior early on due to a lack of accepting and welcoming spaces for LGB people. Mass media and social media also perpetuate SOMs about LGB identities and sexual behavior. Late adolescence and young adulthood include sexual experimentation and peer acceptance. Risky decision-making is amplified and encouraged in rave sub-cultures, giving the impression that it is typical and even expected. This may influence an identification with risky sexual behavior that becomes a self-fulfilling prophecy to maintain negative characteristics of the perceived identity.

“The drugs, the sex, the promiscuity, that’s all the public sees of the gay community because sex sales. Like my cousin who, just between us, is [an] oversexed *hetero[sexual]* cis female, but she makes comments to me like, ‘yeah, I know how y’all get down.’ And I’m like, but I been with my [same sex] girlfriend for like two years now, and she hops from dude to dude. How many times I got to say, ‘We are not all like that!” – Korin [L]

“Gay communities have diligence in taking care of their sexual health, probably more so than a straight person, because in a heteronormative society, straight people are not asked to second guess having sex.” Donaldo [G]

Frustration in their interactions with cisgender heterosexuals who perpetuate hypersexual SOMs in the LGB community seemed to be a part of the territory as an LGB person. Sexual orientation minorities express deep frustration in changing these stereotypes about their sexual behavior. However, most participants felt a responsibility to debunk, or at least not perpetuate the stereotypes. They believed that STI disparities among LGB persons and other sexual orientation minorities could be addressed by changing the public’s perception of the identifying characteristics of the group. As young adults, they rejected the hypersexualized stereotypes about LGB people and understood to question sexual health information that may sound biased or seem to demonize homosexuality.

## Discussion

To gain a deeper understanding of sexual orientation microaggressions (SOMs), the present study utilized reflexive thematic analysis to explore how SOMs shaped LGB young adult’s understanding of sexual health knowledge and attitudes toward sexual risk behavior. Four themes captured the facets of SOMs: (a) early familial moments, (b) religious persecution about their sexuality, with a sub-theme of the intersection of race/ethnicity, (c) reactions to SOMs, with a sub-theme of coping and resilience strategies and sexual health, and (d) sexual education and miseducation, with a sub-theme of sociocultural influences on attitudes toward risky behavior. Furthermore, when reviewing the microaggression taxonomies of Nadal et al. (7), Platt and Lenzen (13), and Kwok and Kwok (11), we found that our findings add detail and additional support to previous themes, such as over-sexualization, derogatory language, assumptions of abnormality, denial of heterosexism, and negative portrayals of LGB identities.

LGB young adults highlighted SOMs tied to early familial moments during adolescence. These SOMs were primarily verbalized by adult family members or adult family friends wishing to impose heteronormative gender norms on them. Some participants mentioned grade school experiences, but parents or extended family members garnered most early SOM messages. This theme aligned strongly with previous literature’s common theme of using heterosexist language and terminology to degrade one’s LGB identity (7, 11, 13) and provides additional details to who is specifically perpetuating this messaging. Furthermore, what made the current study’s theme unique is the continued impact that early familial messaging of heteronormativity had on the development of participants’ sexual and gender identity and how they viewed themselves or internalized homonegative messaging as youth.

Religious persecution around their LGB sexual orientation was developed as a theme and highlighted how the use of religion to control the participants’ sexual orientation and their expression of same-sex attractions impacted their psychological well-being. Regarding previously established SOMs, this theme aligns well with Platt and Lezen’s (13) SOM of Sinfulness, Kwok and Kwok’s (11) SOM of Assumption of Sexual Abnormality, and Nadal et al. (7) Assumption of Sexual Pathology/Abnormality. Specifically, participants discussed how this SOM assumes that homosexuality is not normal and is deserving of religious persecution; which, in turn, seemed to discourage LGB participants’ enjoyment of their sexual development and sexuality, at least during childhood and adolescence.

Furthermore, within the religious persecution theme, a sub-theme was developed, indicating the intersectionality of race/ethnicity within religious persecution. Relatively many African American participants commented on how the “Black church” is particularly harsh about condemnation of homosexuality in their experiences. Other participants discussed how their Western religions were highly heteronormative, and it was used to influence heteronormative behavior expression. However, African American participants spoke about the “Black church” not being welcoming to LGB persons and particularly stressed inflammatory religious condemnations such as “going to hell.” We recognize that this is not representative of all African American church institutions; however, it was recognized as a concern of institutional discrimination in the lived experience among the African American participants in the present study. This sub-theme provides additional and specific details to how there may be cultural differences in how SOMs are expressed in institutional settings and thus warrants further investigation of how racially minoritized LGB young adults experience SOMs and other microaggressions.

To date, research is limited regarding the intersectionality of SOM microaggressions and gender and/or race-ethnicity as they relate to LGB adults. However, identifying as a racial-ethnic or gender minority in addition to a sexual minority status, known as ‘double jeopardy’ (i.e., having two or more minority identities), assumes a higher risk for various victimizations. In fact, women who identify as gay, lesbian, or bisexual are at higher risk for intimate partner violence, for example (42). Another study looked at the intersectionality of race/ethnicity, gender, bilingual status, and racial microaggressions and found that African American male students were significantly different from the other groups (female, White, and Hispanic students) regarding their experiences involving ‘assumptions of inferiority’ microaggressions and school and workplace racial microaggressions (43). Notwithstanding, a review of literature indicated a consensus that future research would benefit from examining the role of gender, sexual orientation, and race-ethnicity as interaction or control variables in future quantitative models examining these topics. We propose that qualitative research may also be focused on this potential intersectionality through purposeful recruitment and methodology (e.g., structured interview scripts) that are developed to explore how race-ethnicity or gender factors into their experiences of SOMs.

In addition to the previously established taxonomies that the current study provides, there were reactions to SOMs, as personal reactions toward SOMs had not been integrated into the systemic perspective of previous taxonomies. We developed two sub-themes of Societal Norms and Coping and Resilience. The sub-theme of Societal Norms highlighted SOMs related to Nadal et al. (7) Discomfort/Disapproval of The LGB Experience SOM and Platt and Lenzen (13) Heterosexist Language/Terminology SOM, as participants discussed their dislike of microaggressions that dismiss their sexual orientation personhood through heterosexist language such as “that’s gay” or not wanting to be ‘bothered’ with the existence of homosexuality. We acknowledged another SOM not previously discussed by the established taxonomies, which was microaggressions related to “outing” their homosexuality to family or friends and the notion of being investigated under suspicion of being gay. Participants shared their coming out experiences being thwarted due to the SOMs expressed by family and community members. Additionally, growing up in a heteronormative society may produce feelings of isolation or confusion as an LGB pre-teen or adolescent recognizes that their sexual orientation does not represent the norm. Thus, ‘coming out’ is a sensitive and personal decision that should not be investigated or prodded by others. In turn, this leads to the suppression of one’s sexual orientation, emotions, and feelings of loneliness that contribute to adverse mental health outcomes (27). Overall sentiments regarding the coming out process revolved around the notion that heterosexual adults do not have to reconcile with their sexual orientation as homosexual adults often do.

In examining their reactions to SOMs, we also developed a sub-theme of Coping and Resilience. Conferring this sub-theme, participants were asked what ideas about sexuality and sexual orientation they would pass on. Responses centered around their resilience to embrace their sexual orientation in a positive way and the rejection of stereotypes present in SOMs. Participants discussed their hopefulness for the next generation. They believe the next generation will be more understanding of their biopsychosocial sexual orientation, and there will be less homonegativity by virtue of their own understanding. This hopefulness can be viewed as a protective factor, distinguishing the present study’s contribution to the SOM taxonomy literature by acknowledging the homo-positivity that developed as a function of their coping and resilience to SOMs. While some participants mentioned the supportive role of their parents or other family members, most emphasized the significance of relying on relationships with friends for support. This is developmentally expected as adolescence is a period where emotional reliance on peers is more evident than reliance on parents; notwithstanding the important protective role of family in the healthy development of one’s sexuality (44, 45). Participants shared how friendships, particularly in high school and college, helped them cope with homophobia and gain acceptance of their sexuality. Being open to sharing was a sentiment shared by most of the LGB participants. However, the present study recognized that this finding may be due to self-selection in study participation.

The theme, Sexual Education and Miseducation included important aspects of previous taxonomies but in the context of sexual health education and attitudes about risky sexual behavior. Kwok and Kwok (11) found a somewhat similar SOM theme, Allowing Institutionally Endorsed Microaggressions, and there were some contributory details from Platt and Lenzen (13) SOM theme, Over-Sexualization, and Nadal et al. (6) SOM theme, Assumption of Sexual Pathology/Abnormality. The present study’s theme delves deeper into understanding how some SOMs related to this taxonomy contained minuscule elements of factual information about sex and disease acquisition, and how this factual information was grossly biased toward heteronormative worldviews and contributed to miseducation. For example, although procreation is the primary biological evolutionary purpose of coitus, there are many other physiological and psychological purposes for engaging in sexual activity. Maslow’s hierarchy of needs and its expansions have consistently listed our physiological need for sex along with other undeniable biological needs such as food and water. Teaching adolescents that sex is only used for procreation denies the normality of sexual awakening and development by introducing confusion and denial, irrespective of sexual orientation; in addition to sexual orientation minorities who must contend with their sexual orientation status within a heteronormative society (46).

Miseducation about sex and sexually transmitted infections (STIs) was prevalent among the group as well. Most notable were the conversations surrounding HIV still being a ‘gay man’s disease,’ as it denies that unprotected sex exacerbates the spread while blaming a decision to express a natural state of same-sex attraction. Miseducation also centered around oral sex as being a safer alternative to penetrative sex. Some participants thought that oral sex exchanges less fluids than penetrative sex and, therefore, was less likely to result in infection transference. However, young adults may fail to realize that disease infection occurs on a microscopic level through viruses and bacteria in many instances. Therefore, unprotected oral sex can be just as problematic in efforts to reduce STI incidence, just as unprotected penetrative sex does.

Although sociocultural influences on attitudes toward risky sexual behavior emerged as its own theme, some of its thematic discussion points were intertwined with the miseducation about sexual health. Drugs and sex were discussed together as being the “lifestyle” within the gay culture, particularly rave culture. This piece of miseducation fails to amplify the deleterious effects of drugs while simultaneously associating them with sexual happiness. Drugs may also serve to alter decision-making, making them more susceptible to engaging in risky sexual behavior, thinking it is part of the norm in these environments (47).

Another example of this miseducation is that HIV gained much-needed attention in the 1980s primarily due to the disease disproportionately presenting among gay males who engaged in risk-related anal sex. However, this does not and should not translate into AIDS being a “gay man’s disease,” which serves to miseducate those who believe that heterosexual or lesbian persons are at significantly less risk due to their non-participation in anal sex, as there are many other psychosocial risk and protective factors to consider. Particularly, attitudes about sexual risk corroborated Formby (48), whose findings support misinformation regarding women who have sex with women.

Socio-culture played a role in their attitudes about sex and STI risk and was developed as a sub-theme within the Sexual Education and Miseducation theme. Attitudes about sexual health within the LGB community have notoriously been shaped by their vulnerabilities to STIs compared to their heterosexual counterparts. Despite the expanding literature highlighting barriers that disproportionately affect the sexual health of LGB adults, there remains a crucial need to comprehend how the barriers impact their sexual health knowledge and attitudes. Vulnerabilities to STIs have notoriously shaped sexual health within the LGB community in comparison to their heterosexual counterparts. Some participants even experienced the desire to change their inherent attractions to adhere to heteronormative standards by engaging in heterosexual sex reluctantly to ‘prove a point,’ so to speak. Furthermore, reluctant sex would not constitute a healthy sexual relationship.

Rave culture stereotypes may be conceptualized as a sociocultural barrier as some participants discussed that they were introduced to rave culture stereotypes as a standard of behaving upon understanding their LGB group status. Nevertheless, as the young adults represented in the current study grew throughout adolescence, they began to understand the fallacy of mass media stereotypes and reject the label of oversexualization. There was a consensus that young adults who are a part of the LGB community realize much of the mass media portrayal of sexuality was a myth and often assumed a forced heteronormative agenda, although this may not have been their initial impressions of LGB culture. In contrast, some believed that the over-sexualization stereotypes make them “more diligent” about their sexual health. However, we must point out that this reflects our participants’ experiences and may not be generalizable to all LGB young adults.

The use of reflexive thematic analysis allowed us to locate the participants and their data within the wider sociocultural and historical context. Participants in the study discussed their encounters with SOMs, detailing experiences involving sexual orientation slurs, religious persecution, disregard for non-heterosexual behavior within family environments, and emotional reactions such as anger, apathy, and self-repression in response to SOMs. While blatant and dismissive heteronormative microaggressions were expected given the discriminatory history of LGB persons, family interactions are particularly highlighted as impactful, even if the SOM comment by family members was not directed to the LGB person. This may give an LGB person inherent views on the intolerances in society surrounding sexual attraction and orientation.

### Strengths and limitations

Our ongoing research intends to demonstrate the connection between microaggressions and sexual health behavior and identify ways to build resilience and environmental strongholds that will help this sub-population maintain positive sexual health and reduce sexual health disparities. Continuing to integrate the lived experiences of LGB people qualitatively may aid in developing clinical interventions and policies that support the mental and physical health of the community. Strengths of this study included all self-identified racial-ethnically diverse LGB participants. Constructivism, as the theoretical reference for the present study, does not strictly adhere to the philosophy of objectivism and maintains an understanding that quantitative approximations with variable degrees of accuracy are not the only way to access the truth about the natural world. Notwithstanding, the information attained in the present study may lend insights into testable hypotheses for future research.

Although suitable for the present study’s qualitative analysis, our study’s sample size was relatively small, which limits the extent the study’s findings can be generalized to a broader LGB context. Furthermore, recruitment occurred at a single university campus, which may not adequately capture perspectivists that exist outside of this setting and introduce sample bias. However, it is important to note that while these participants were from a single minority-serving university campus, it is likely that they brought diversity in their experiences and identity given that participants may not be from the same regional area prior to attending university. Lastly, as we are mindful of our identity and lived experiences and their influence on this study, it is important to note that the goal of reflexive thematic analysis is to integrate these experiences, thoughts, and assumptions into the construction of themes, as we are not free of bias (49, 50). Therefore, the present study is context-dependent and limits generalizability to other LGB people without further inquiry.

### Implications and future research

Taken together, the study’s findings have extended established SOM taxonomies by providing insight into how these categorized experiences could shape the attitudes and knowledge about sexual health among LGB adults. Collectively, participants acknowledged the influence of gay culture, both past and present, in shaping not only their sexuality but also their behaviors, such as condom use. This extension provided a deeper understanding of the nuances between SOMs and their impact on the sexual health perceptions of LGB adults.

Supportive family and friends seemed to buffer adverse sociocultural effects, as quality relationships may provide essential emotional support, validation, and a sense of community for navigating the complexity of sexuality and avoiding sexual risk. Interestingly, aside from avoidance or denial, other maladaptive coping strategies (e.g., promiscuous sex, dangerous sex, excessive drug use, etc.) were not a central theme in any focus group discussions. While the findings limit generalizability, participants reasonably represented a diverse group of sexually responsible LGB adults who debunked the myth of rampant sexual promiscuity among LGB adults. Moreover, discussions surrounding prevalent myths and misconceptions related to HIV and STI testing underscored the need for informed and accurate knowledge in these areas. Communities must work together and aspire to establish norms promoting healthy mental and developmental adjustment throughout the life course, irrespective of sexual orientation.

To our knowledge, this is the first study to examine SOMs as they relate to LGB sexual health and attitudes toward risky behaviors. These insights offer valuable information for advocating for educational policy changes aimed at establishing an inclusive and accepting environment that provides accurate sexual health information. Addressing the existing gaps in access to sexual health resources is crucial. Given the presence of anti-LGB legislation today, treating LGB individuals as inherently “at risk” simply due to their sexual orientation status and implementing discriminatory policies complicates the issue, making it challenging to receive adequate and affirming sexual health knowledge and resources (51).

Despite efforts to improve diversification in the workplace, minorities remain underrepresented in corporate and legislative leadership and institutions that could dismantle microaggression-latent cultures within their work environments. Holder et al. (60)concluded, from their qualitative study examining microaggressions against Black women in corporate leadership, that despite being in high-achieving positions of power, Black women consistently contended with racial microaggressions involving invisibility, exclusion, and an assumed universality of the Black experience. Although not explicit to their sexual orientation, experiences involving these same themes were found among some of the LGB participants in the present study. Greater minority diversity in essential institutions (e.g., government, corporate workplace, school, hospitals, etc.), coupled with Allies in positions of power and an understanding of microaggressions at a systematic level are foundational elements needed to dismantle microaggression-latent cultures.

Additionally, studies have shown the benefits of LGB inclusive sexual education reducing adverse mental health symptoms such as depression and suicidal ideation, and experiences of bullying (52), thus the results can also inform the development of a comprehensive sexual health curriculum tailored to the needs of LGB youth and adults to support their physical and mental well-being. Many participants had avoidant and/or upsetting reactions to SOMs that pointed to denying their sexuality as youth. This hinders the exchange of important sexual health information at a time when adolescents and young adults are more curious and impressionable. Indeed, previous research has alluded to the lack of factual knowledge, particularly on the part of sex education high school teachers, for the paucity of LGB(TQ) sexual health issues being discussed in current sex education curricula (53). Because it has been suggested that our traditional notion of sex education in public schooling emerged from a social fear of adolescent sexuality impinging on society’s social hygiene (54), social scientists and medical experts must continue to advocate for more inclusive and comprehensive understandings of human sexuality, biologically and socially. This may be difficult, but strides and actualization of this grand and beneficial inclusion can be made. Primary education is an excellent entry into shaping a cultured society that reduces negative experiences among LGB adolescents and young adults. Sex education curricula should adopt mission statements and anti LGB(TQ) nondiscrimination policies that would solidify their protection against retaliation for simply “being.” Additionally, training on these issues for school faculty and staff, incorporating sexual minority civil rights in history curricula, developing sexual minority Allies, and implementing micro-intervention (−interventions created to specifically understand and reduce microaggressions) are needed (55–57).

Furthermore, future research regarding SOMs experienced in healthcare settings may provide insight into LGB youth and adults’ experiences with concealing their sexual orientation, STI testing practices, and receiving sexual health knowledge and resources. Community health centers, including university health centers serving students, should be attentive to sexual and gender minority concerns about receiving care such as fear and shame from society. Many of the implements recommended for champion sex education curricula, such as specially trained staff, inclusive mission statement and vision, and specific anti-discrimination policies, are also relevant to university health centers and the university’s academic affairs departments well.

Given the present study’s findings, future research should also examine the intersectionality of race-ethnicity, gender, and LGB orientation as it relates to understanding sexual health knowledge and attitudes. This perspective is essential for developing interventions, sexual health knowledge, and policies that address the unique challenges faced by racial-ethnic and gender minoritized LGB youth and adults. Empirically investigating this intersectionality can allow us to fully understand the complexities of interconnected, sociocultural, and sociopolitical identities (58). Research has provided excellent recommendations on the conceptualization and measurement of the intersectionality of race-ethnicity and/or gender and SOMs, including an accepted understanding of what is meant by *intersectionality*, the importance of real-world experiences, and a framework for a more accurate and comprehensive racial ethnic and sexual gender microaggressions measures.

## Conclusion

The examination of SOMs constructed through the present study included the invisibility or denial of same-sex relationships, the portrayal of HIV as an LGB-only issue, the inclusion of racial-ethnic LGB experiences, and the dissemination of miseducation related to sexuality and coitus. The study’s findings can provide a foundation for future studies to explore the development of other taxonomies, including the intersectional experiences (e.g., racial and sexual orientation microaggressions in the LGB community) and their relation to sexual behavior. Various mental health and sexual health risk and protective factors can be ascertained from the present study. Unanswered inquiries extending from this research may involve how SOM experiences are clustered within LGB young adults by family characteristics (e.g., varying religions, parents’ education, family composition, etc.), and how this influences mental health and sexual health risk factors, among others. A deeper exploration is warranted.

Growing up in a heteronormative society does not equate to homonegativity. Being homosexual or bisexual in a heteronormative society allowed our participants to be more open and accepting of others’ sexuality, regardless of gender orientation and sexual orientation expression. Moreover, we support the sentiment that, moving forward, society will continue to understand that “your sexuality is yours and yours alone.”

## Data Availability

The datasets presented in this article are not readily available because raw data may contain identifiable descriptions. Requests to access the datasets should be directed to ccarmack@central.uh.edu.
